# FapR: From Control of Membrane Lipid Homeostasis to a Biotechnological Tool

**DOI:** 10.3389/fmolb.2016.00064

**Published:** 2016-10-06

**Authors:** Daniela Albanesi, Diego de Mendoza

**Affiliations:** Laboratorio de Fisiología Microbiana, Instituto de Biología Molecular y Celular de Rosario, Consejo Nacional de Investigaciones Científicas y Técnicas, Universidad Nacional de RosarioRosario, Argentina

**Keywords:** lipid synthesis, FapR, transcriptional regulation, Gram-positive bacteria, *in vivo* malonyl-CoA sensor, synthetic biology

## Abstract

Phospholipids and fatty acids are not only one of the major components of cell membranes but also important metabolic intermediates in bacteria. Since the fatty acid biosynthetic pathway is essential and energetically expensive, organisms have developed a diversity of homeostatic mechanisms to fine-tune the concentration of lipids at particular levels. FapR is the first global regulator of lipid synthesis discovered in bacteria and is largely conserved in Gram-positive organisms including important human pathogens, such as *Staphylococcus aureus, Bacillus anthracis*, and *Listeria monocytogenes*. FapR is a transcription factor that negatively controls the expression of several genes of the fatty acid and phospholipid biosynthesis and was first identified in *Bacillus subtilis*. This review focuses on the genetic, biochemical and structural advances that led to a detailed understanding of lipid homeostasis control by FapR providing unique opportunities to learn how Gram-positive bacteria monitor the status of fatty acid biosynthesis and adjust the lipid synthesis accordingly. Furthermore, we also cover the potential of the FapR system as a target for new drugs against Gram-positive bacteria as well as its recent biotechnological applications in diverse organisms.

## Introduction

**Figure 1:**
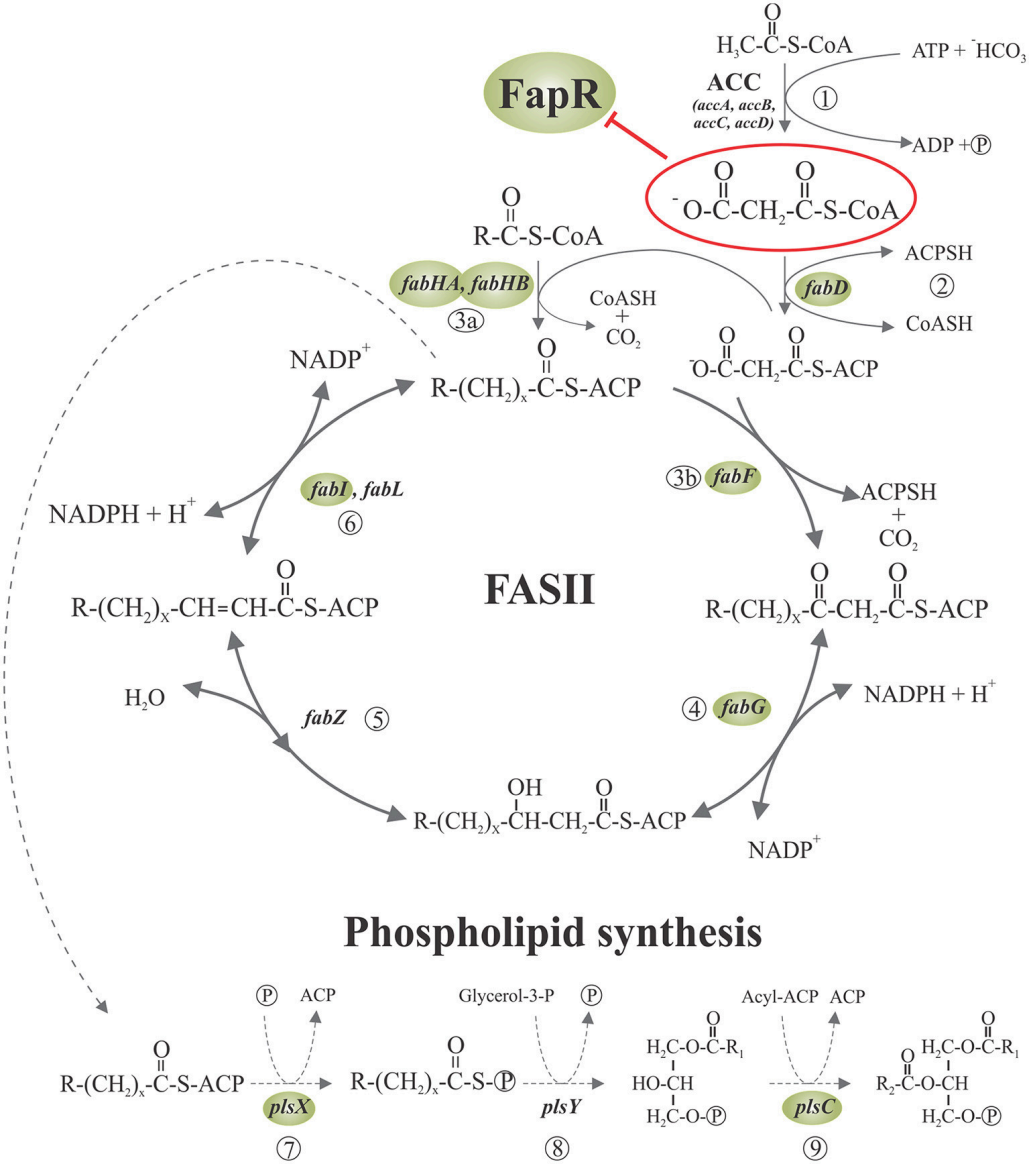
**Fatty acid synthesis and phospholipid initiation steps in *Bacillus subtilis***. Malonyl-CoA is generated from acetyl-CoA by acetyl-CoA carboxylase (ACC) (1) and then is transferred to ACP by malonyl-CoA transacylase (2). The FabH condensing enzymes initiates the cycles of fatty acid elongation by condensation of acyl-CoA primers with malonyl-ACP (3a). The resultant β-ketoester is reduced by the β-ketoacyl-ACP reductase (4). Then, the β-hydroxyacyl-ACP is dehydrated to the trans-2 unsaturated acyl-ACP by β-hydroxyacyl-ACP dehydrase (5), which is finally reduced by enoyl reductase (6). Subsequent rounds of elongation are initiated by the elongation-condensing enzyme FabF (3b) to generate an acyl-ACP two carbons longer than the original acyl-ACP at the end of each cycle. The long chain acyl-ACP end products of fatty acid synthesis are transacylated in three steps to glycerolphosphate, to generate phosphatidic acid (PA), a key intermediate in the synthesis of phospholipids. First, PlsX catalyzes the synthesis of fatty acyl-phosphate from acyl-ACP (7); then, PlsY transfers the fatty acid from the activated acyl intermediate to the 1-position of glycerol-3-phosphate (8) and finally, lyso-PA is acylated to PA by PlsC (9). Expression of the genes surrounded by shaded ellipses is repressed by the transcriptional regulator FapR, whose activity is, in turn, antagonized by malonyl-CoA (enclosed in a red ellipse). R denotes the terminal group of branched-chain or straight-chain fatty acids. Adapted from Albanesi et al. (2013).

The cell membrane, consisting mainly of a fluid phospholipid bilayer in which a variety of proteins are embedded, is an essential structure to bacteria making membrane lipid homeostasis a crucial aspect of bacterial cell physiology. The production of phospholipids requires of the biosynthesis of fatty acids and their subsequent delivery to the membrane-bound glycerol-phosphate acyltransferases. In all organisms fatty acids are synthetized via a repeated cycle of reactions involving the condensation, reduction, hydration, and reduction of carbon-carbon bonds (Rock and Cronan, 1996; Campbell and Cronan, 2001). In mammals and other higher eukaryotes, these reactions are all catalyzed by a large multifunctional protein, known as type I synthase (FAS I), in which the growing fatty acid chain is covalently attached to the protein (Rock and Cronan, 1996; Campbell and Cronan, 2001). In contrast, bacteria, plant chloroplasts, and *Plasmodium falciparum* contain a type II system (FAS II) in which each reaction is catalyzed by a discrete protein. A characteristic of FASII is that all fatty acyl intermediates are covalently connected to a small acidic protein named acyl carrier protein (ACP), and sequentially shuttled from one enzyme to another. A key molecule for fatty acid elongation is malonyl—coenzyme A (CoA) which is formed by carboxylation of acetyl-CoA by the enzyme acetyl-CoA carboxylase (ACC) (Figure 1). This biosynthetic scheme is conserved in all fatty acid producing bacteria, but the substrate specificity of some of the enzymes involved in the pathway leads to the variety of fatty acids found in different bacterial genera (Campbell and Cronan, 2001; Lu et al., 2004). When the acyl-ACPs reach the proper length they become substrates for the acyltransferases that transfer successively the fatty acyl chains into glycerol phosphate to synthetize phosphatidic acid (PtdOH), the universal intermediate in the biosynthesis of membrane glycerophospholipids (Figure 1: Campbell and Cronan, 2001; Rock and Jackowski, 2002). There are two enzyme systems that carry out the first transacylation reaction in bacteria. In the first one, present exclusively in Gram-negative bacteria (primarily gamma-proteobacteria), either acyl-ACP or acyl-CoA thioesters are utilized by the membrane-bound PlsB acyltransferase to acylate position 1 of glycerol-P giving 1-acylglycerol phosphate (Parsons and Rock, 2013). The second enzyme system, widely distributed and predominating in Gram-positive bacteria, consist of the PlsX/Y pathway for 1-acyl-glycerol phosphate formation (Lu et al., 2006; Schujman and de Mendoza, 2006; Paoletti et al., 2007). PlsX is a membrane associated protein (Sastre et al., 2016) that catalyzes the formation of a novel acyl donor, acyl phosphate (acyl-P), from acyl-ACP. This activated fatty acid is then used by the membrane-bound PlsY acyl transferase to acylate the position 1 of glycerol phosphate. The PlsX/PlsY system is also present in *E. coli* although its precise role is still an enigma as *plsB* is an essential gene in this bacterium (Parsons and Rock, 2013). Independently of the first enzyme system used, the second acyl transferase in PtdOH formation is PlsC, which is universally expressed in bacteria. This enzyme completes the synthesis of PtdOH by transferring an acyl chain to the position 2 of 1-acyl-glycerol phosphate. In the case of Gram-positive bacteria, PlsC isoforms exclusively utilize acyl-ACP (Lu et al., 2006; Paoletti et al., 2007), while *E. coli* PlsC can use both, acyl-ACP or acyl-CoA, as substrates (Coleman, 1992).

The fluidity of the lipid bilayer is essential for the normal function of the cellular membrane and bacteria normally control its physical state by modifying the incorporation of a mixture of fatty acids with different melting temperatures into phospholipids. In this sense, many bacteria respond to a decrease in temperature, which increases membrane rigidity, by increasing the proportion of unsaturated fatty acids (UFAs) into the phospholipids and viceversa (Zhang and Rock, 2008). Unsaturated double bonds in lipids generate kinks into the otherwise straightened acyl hydrocarbon chain and thereby increase membrane fluidity. Hence, the production of UFAs and its regulation are important processes in membrane homeostasis in bacteria and the underlying diverse mechanisms have been recently revised elsewhere (Mansilla et al., 2008; Parsons and Rock, 2013).

Due to the fact that the membrane lipid bilayer is an essential structure for every living cell and its biogenesis implies a high energetic cost, mainly due to fatty acid biosynthesis, organisms have developed a variety of homeostatic mechanisms to finely adjust the concentration of lipids at particular levels. Bacteria possess regulatory mechanisms acting directly on the activities of the lipid biosynthetic enzymes, but have also evolved sophisticated mechanisms to exert an exquisite control over the expression of the genes involved in lipid metabolism (Zhang and Rock, 2008; Parsons and Rock, 2013). Six transcriptional regulators controlling the expression of genes involved in fatty acid biosynthesis have been identified to date in bacteria. Among them, FadR (Henry and Cronan, 1991, 1992; Lu et al., 2004), DesR (Aguilar et al., 2001; Mansilla and de Mendoza, 2005), FabR (Zhang et al., 2002), and DesT (Zhu et al., 2006; Zhang et al., 2007), are committed to adjust unsaturated fatty acids to proper levels in membrane phospholipids while FapR (Schujman et al., 2003) and FabT (Lu and Rock, 2006) are global transcriptional repressors in Gram-positive bacteria that simultaneously regulate the expression of a number of genes involved in fatty acid and phospholipid metabolism.

This review focuses on the genetic, biochemical and structural characterization of FapR which paved the way to a major advance in our understanding of the molecular basis of the lipid homeostasis control in bacteria. We will also cover the potential of this regulatory system as a target for new antibacterial compounds as well as emerging biotechnological applications based on it.

## The discovery of the FapR system

FapR from *Bacillus subtilis* was the first global transcriptional regulator of FASII to be discovered in bacteria (Schujman et al., 2003). The initial evidence that fatty acid biosynthesis was transcriptionally regulated came from the study of *lacZ* fusions to the promoter region of the *fabHAF* operon of *B. subtilis*, which codes for two key enzymes involved in the elongation of fatty acids (Schujman et al., 2001). These studies showed that the operon *fabHAF* is transcribed during exponential phase but when the cell culture approaches to stationary phase its transcription is turned off (Schujman et al., 2001). This finding is consistent with the observation that during exponential growth bacteria constantly produce new membrane in order to divide and hence need to actively synthetize fatty acids. Nevertheless, when cell division is completed membrane growth stops and fatty acid synthesis is turned off. An important finding was that when fatty acid synthesis is inhibited the transcription of the *fabHAF* operon is induced with the concomitant increment in protein levels (Schujman et al., 2001). Thus, it was proposed that *B. subtilis* is able to detect a decrease in the activity of FASII and respond accordingly by inducing the production of the condensing enzymes FabHA and FabF (Schujman et al., 2001). Moreover, DNA microarray studies indicated that upon inhibition of fatty acid synthesis the transcription of ten genes was induced (Schujman et al., 2003). These genes coded for proteins involved in fatty acid and phospholipid biosynthesis and belonged to six operons (the *fap* regulon) (Schujman et al., 2003). Furthermore, a conserved 17 bp inverted repeat within, or immediately downstream, of the *fap* predicted promoters, consistent with a putative binding site for a transcriptional repressor, was identified (Schujman et al., 2003). The corresponding binding protein was isolated from cells extracts using a DNA fragment carrying the promoter region of *fabHA* and identified by N-terminal sequencing (Schujman et al., 2003). The gene encoding the global transcriptional repressor was named *fapR* for *fatty acid and phospholipid regulator* (Schujman et al., 2003). The binding of FapR to the promoter regions of the regulated genes, and its dependence on the 17 inverted repeats was demonstrated *in vitro*. It was also showed that in a *fapR* null mutant the expression of the *fap* regulon is upregulated and that this expression is not further increased upon inhibition of FASII (Schujman et al., 2003). Therefore, it was established that FapR was a novel global negative regulator of lipid biosynthesis in Gram-positive bacteria and that FapR was involved in the observed induction of transcription in the presence of fatty acids synthesis inhibitors (Schujman et al., 2003). Bioinformatic analyses indicated that FapR is present and highly conserved in all the species of the *Bacillus, Listeria*, and *Staphylococcus* genera (all including important human pathogens like *Bacillus anthracis, Bacillus cereus, Listeria monocytogenes*, and *Staphylococcus aureus*) as well as in the pathogen *Clostridium difficile* and other related genera. However, *fapR* was not found in Gram-negative bacteria or other Gram-positive genera (Schujman et al., 2003). Furthermore, in the bacterial species bearing FapR, the consensus binding sequence for the repressor is also highly conserved in the putative *fapR* promoter region. Altogether, the observations suggested that the regulatory mechanism identified in *B. subtilis* could be conserved in many other bacteria (Schujman et al., 2003). Indeed, genetic and biochemical assays proved this is the case in *S. aureus* (Albanesi et al., 2013).

## Malonyl-CoA: The effector molecule

A central question in the regulation of the *fap* regulon by FapR was how the status of fatty acids synthesis controlled the activity of the repressor. The fact that (i) the *acc* genes, encoding the subunits of the acetyl-CoA carboxylase (ACC), which catalyzes the synthesis of malonyl-CoA (Figure 1), are not under FapR control (Schujman et al., 2003), (ii) malonyl-CoA concentrations are known to increase upon inhibition of fatty acid synthesis (Heath and Rock, 1995), and (iii) the only known fate of malonyl-CoA in *B. subtilis* and most other bacteria is fatty acid synthesis (James and Cronan, 2003), pointed to malonyl-CoA as a reasonable candidate to be the regulatory ligand. Two observations gave experimental support to this hypothesis. First, expression of the *fap* regulon was derepressed by antibiotics that inhibit fatty acid biosynthesis with the concomitant increase in the intracellular levels of malonyl-CoA (Schujman et al., 2001). Second, this upregulation was abolished by precluding the transcription of genes encoding the subunits of the acetyl-CoA carboxylase (ACC) (Schujman et al., 2003).

A key issue was to establish if malonyl-CoA bound directly to FapR to regulate its activity or if it was first converted into another product that acted as a signaling molecule. The finding that antibiotics against different steps of FASII led to the transcriptional induction of the *fap* regulon, even when the *B. subtilis fabD* gene (Morbidoni et al., 1996) was not expressed, suggested that malonyl-CoA could be the direct effector of FapR (Schujman et al., 2003). FabD converts malonyl-CoA into malonyl-ACP, which, in turn, is only utilized in the elongation of fatty acid synthesis (de Mendoza et al., 2002). *In vitro* transcription experiments from several promoters of the *fap* regulon, including the *fapR*-operon promoter (P*fapR*), proved that FapR is unable to repress transcription in the presence of malonyl-CoA. Moreover, these assays showed that this molecule operates not only as a direct but also as a specific inducer of the *fap* promoters since different acyl-CoA derivatives related to malonyl-CoA (such as acetyl-CoA, propionyl-CoA, succinyl-CoA, and butyryl-CoA), were not able to prevent FapR transcriptional repression (Schujman et al., 2003). The same direct and specific role of malonyl-CoA as the effector molecule was shown for FapR of *S. aureus* (*Sa*FapR) (Albanesi et al., 2013).

## Structural snapshots of the FapR regulation cycle

**Figure 2:**
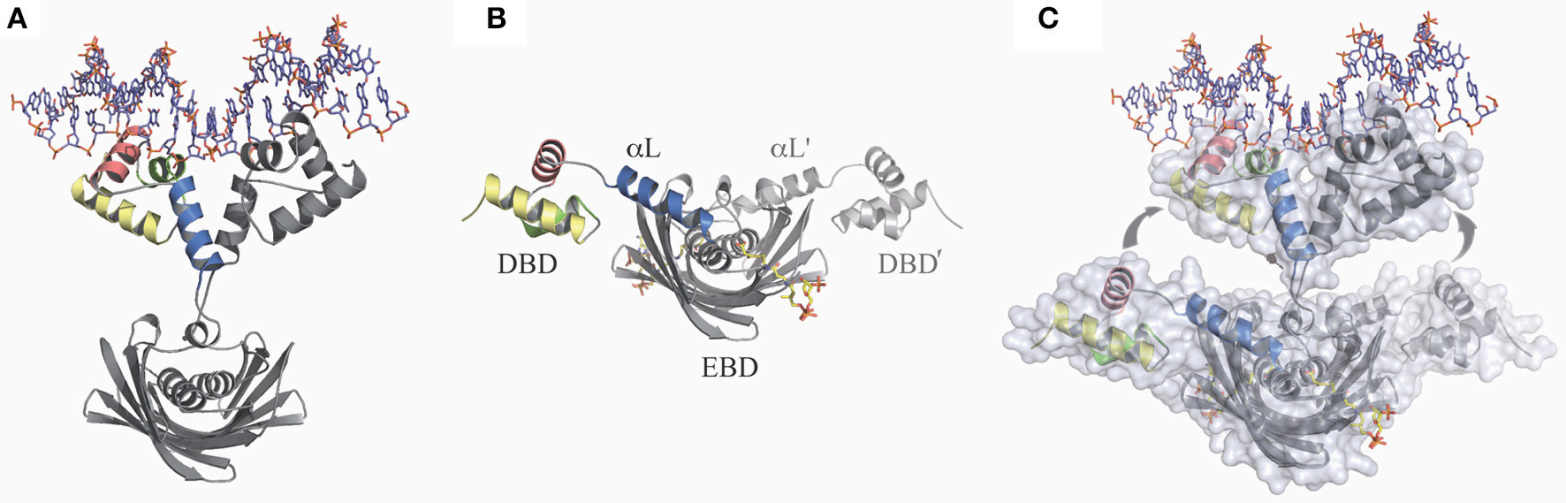
**The transitional switch between the relaxed and tense states of FapR involves a significant structural rearrangement of the DBDs**. **(A)** Relaxed state: FapR in complex with DNA in which the amphipathic linker α-helix (αL) from each protomer associates with each other. **(B)** Tense state: FapR in complex with malonyl-CoA (shown in stick representation). **(C)** Superposition of the two conformational states of the repressor illustrating the structural transition which involves substantial changes and large (~30 Å) inter-domain movements. Solvent accessible surfaces are shown in transparent to highlight the DNA-induced dissociation of the invariant effector-binding domain (EBD) from the DNA-binding domains (DBDs). The molecules are shown in light (relaxed) and dark (tense) gray, except for the helices from one DBD (colored). Adapted from Albanesi et al. (2013).

Like many transcriptional regulators in bacteria, FapR is a two-domain protein with an N-terminal DNA-binding domain (DBD) connected through a linker α-helix (αL) to a larger C-terminal effector-binding domain (EBD) (Schujman et al., 2006). The first insights on the molecular mechanism for the control of FapR activity came from the crystal structures of truncated forms of FapR from *B. subtilis* (*Bs*FapR) (Schujman et al., 2006). These structures showed that the EBD is a symmetric dimer displaying a “hot-dog” architecture, with two central α-helices surrounded by an extended twelve-stranded β-sheet (Schujman et al., 2006). This fold is similar to the one observed in many homodimeric acyl-CoA-binding enzymes (Leesong et al., 1996; Li et al., 2000) involved in fatty acid biosynthesis and metabolism (Dillon and Bateman, 2004; Pidugu et al., 2009). Interestingly, FapR, a bacterial transcriptional repressor, seems to be the only well-characterized protein to date with no-enzymatic function that harbors the “hot-dog” fold (Albanesi et al., 2013). On the other hand, the EBD domain of *Bs*FapR was crystallized in complex with malonyl-CoA. Comparison of both structures revealed structural changes induced by the effector molecule in some ligand-binding loops of the EBD that were suggested to propagate to the N-terminal DBDs impairing their productive association for DNA binding (Schujman et al., 2006). However, the actual mechanisms involved in the regulation of FapR activity remained largely unknown due to the lack of detailed structural information of the full-length repressor and its complex with DNA. Recently, important mechanistic advances into the mode of action of FapR were done through the structural characterization of the full-length repressor from *S. aureus* (*Sa*FapR). The crystal structures of *Sa*FapR were obtained for the protein alone (apo-*Sa*FapR) as well as in complex with the cognate DNA operator and the effector molecule malonyl-CoA (Albanesi et al., 2013) (Figure 2).

### Structure of the *Sa*FapR-DNA complex

The crystal structure of the *Sa*FapR-DNA complex was obtained using a 40-bp oligonucleotide comprising the P*fapR* promoter, which, as mentioned above, belongs to the *fap* regulon (Schujman et al., 2006). In the crystal, two *Sa*FapR homodimers were observed to bind to each DNA molecule. Interestingly, an inverted repeat covering half of the FapR-protected region in DNAseI footprinting analyses (Schujman et al., 2006), corresponded to the recognition site of one of the homodimers (Albanesi et al., 2013). This suggested a sequential mechanism of binding that was confirmed by isothermal titration calorimetry (ITC) studies of the *Sa*FapR-DNA interaction, which also provided the dissociation constants of each binding reaction (Albanesi et al., 2013). In the crystal structure of the *Sa*FapR-DNA complex, each protein homodimer exhibited an elongated asymmetric conformation with the two DNA-bound DBDs completely detached from the central dimeric “hot-dog” EBD (Figure 2A) (Albanesi et al., 2013). In each homodimer the amphipatic linker α-helixes from the protomers (αL and αL′) interact, mainly through their exposed hydrophobic faces, playing an important role in the stabilization of *Sa*FapR's molecular architecture in the complex with DNA (Figure 2A) (Albanesi et al., 2013). On their hand, both DBDs interact in a similar manner with DNA establishing sequence-specific contacts between the helix-turn-helix motifs with the major and minor grooves of the DNA double helix (Albanesi et al., 2013). Importantly, two arginine residues from each linker αL (one from αL and one from αL′) make base-specific interactions in the minor groove promoting its opening and inducing a pronounced local bending of DNA (Albanesi et al., 2013). Notably, the aminoacid residues making key contacts with DNA are highly conserved in FapR from all bacterial species where it was identified indicating the DNA-binding-mode of this transcriptional repressor is conserved (Albanesi et al., 2013).

### Structure of the *Sa*FapR-malonyl-CoA complex

The crystal structure of full-length *Sa*FapR in complex with malonyl-CoA showed that in the presence of the effector molecule the repressor adopts a quaternary arrangement that is different and more compact than when bound to DNA (Figure 2B) (Albanesi et al., 2013). In this conformation, both amphipathic linker helices αL bind to either side of the central EBD domain instead of interacting with each other as when binding DNA. Like this, the two DBDs domain are far apart from each other, resulting in a non-productive conformation incompetent to bind DNA. Stabilization of the observed quaternary organization of the protein is principally due to the interaction of the linker αL with the lateral face of the EBD (Albanesi et al., 2013). Concerning ligand binding, the structure showed that a tunnel is formed at the interface between the two protomers in the *Sa*FapR homodimer into which the phosphopantetheine group is bound, adopting the same conformation as observed in the truncated *Bs*FapR-malonyl-CoA complex structure, as well as in a number of acyl-CoA-binding proteins harboring the “hot-dog” fold (Albanesi et al., 2013). In this way, the ligand malonate is completely occluded from the bulk solvent. The charged carboxylate group of malonate is neutralized at the bottom of the binding pocket by a specific interaction with an arginine residue. Upon engagement of this arginine in effector binding, a local reorganization is triggered that ultimately leads to surface reshaping and stabilization of the non-productive conformation, thus preventing DNA binding (Albanesi et al., 2013). On the other hand, the adenosine-3′-phosphate moiety of malonyl-CoA is largely exposed to the solvent making no specific contacts with the protein. This implies that *Sa*FapR specifically recognizes the malonyl-phosphopantetheine moiety of the ligand (Albanesi et al., 2013) in agreement with the fact that either malonyl-CoA or malonyl-acyl carrier protein (malonyl-ACP) can both function as effector molecules (Martinez et al., 2010). A detailed comparison of the complexes of full-length *Sa*FapR and the truncated form of *Bs*FapR (lacking the DBDs) with malonyl-CoA revealed a conserved structural arrangement of the EBD core and ligand binding effects. Altogether, the structural alignment indicates an identical mode of malonyl-CoA binding and also the conservation of the DBD–αL–EBD interactions required to stabilize the FapR-malonyl-CoA complex as observed in the *Sa*FapR model (Figure 2B) (Albanesi et al., 2013).

### The structure of full-length *Sa*FapR

Full-length *Sa*FapR was also crystallized in the absence of ligands (apo-*Sa*FapR) and two crystals forms were obtained (Albanesi et al., 2013). In the different structures, most of the crystallographic independent repressor protomers exhibited the non-productive quaternary arrangement with helix αL bound to the lateral face of the EBD, as observed in the structure of *Sa*FapR in complex with malonyl-CoA (Figure 2B), strongly suggesting that in solution the apo-protein would also display this conformation (Albanesi et al., 2013). However, in one of the crystal forms, the helix αL and the associated DBD of one *Sa*FapR protomer could not be modeled due to their high flexibility and the corresponding first visible residues connecting the helix αL with the EBD exhibited a similar conformation to that found for one subunit of the repressor in the asymmetric *Sa*FapR-DNA complex (Figure 2A) (Albanesi et al., 2013). These facts and other crystal parameters (like the extensive crystal contact engagement, the high temperature factors or even the partial disorder displayed by the helix-turn-helix motifs) suggested that alternative conformational states of *Sa*FapR, marked by flexible DBDs, would coexist in solution (Albanesi et al., 2013).

### Structural transitions along the FapR regulation cycle

The structural snapshots of full-length *Sa*FapR along its regulation cycle revealed distinct quaternary arrangements for the DNA-bound (relaxed) and the malonyl-CoA-bound (tense) forms of the repressor, with the linker αL involved in different protein-protein interactions in each case, highlighting a functional switch entailing a large-scale structural rearrangement (Figure 2C) (Albanesi et al., 2013). Indeed, the amphipathic αL, that in the tense state binds through its hydrophobic face to the protein EBD (Figure 2B), dissociates and moves ~30 Å to finally interact with αL from the second protomer (αL′) and with DNA in the relaxed state (Figure 2A) (Albanesi et al., 2013). Furthermore, the structural analysis of apo-*Sa*FapR in two distinct crystal forms also showed that the ligand-free repressor species can populate both, the tense and relaxed conformational states (Albanesi et al., 2013). This suggested that DNA would promote and stabilize the relaxed form of the repressor while an increment in the intracellular concentration of malonyl-CoA would not only trigger the structural changes leading to disruption of the repressor-operator complex but would also drive a shift of the ligand-free *Sa*FapR population toward the tense form (Albanesi et al., 2013).

## The FapR system as a target for new antibacterial drugs

As discussed above, bacterial fatty acid biosynthesis is essential for the formation of biological membranes. Indeed, the importance of the pathway in bacterial physiology is highlighted by the existence of multiple natural products that target different points in this biosynthetic route (Parsons and Rock, 2011). The emergence of resistance to most clinically deployed antibiotic has stimulated considerable interest in finding new therapeutics, leading to a significant effort in academia and industry to develop antibiotic that target individual proteins in fatty acid biosynthesis. One concern about such drugs is that fatty acids are abundant in the mammalian host, raising the possibility that fatty acid synthesis inhibitors would be bypassed *in vivo* (Brinster et al., 2009). Although all bacteria studied to date are capable of incorporating extracellular fatty acids into their membranes, recent research shows that, opposite to what happens in *Streptococcus pneumoniae* (Parsons et al., 2011), exogenous fatty acids cannot circumvent the inhibition of FASII in *S. aureus* and many major human pathogens (Yao and Rock, 2015).

Notably, disruption of FapR-malonyl-CoA interactions by structure-based amino acid substitutions in *S. aureus* leads to permanent repression of fatty acid and phospholipid synthesis, which is lethal and cannot be overcome by addition of exogenous fatty acids (Albanesi et al., 2013), as observed with antibiotics targeting FASII (Parsons et al., 2011). Thus, the distinctive mode of action of FapR together with the promising *in vivo* results highlight lipid homeostasis and the FapR system as a propitious target for the development of new drugs against Gram-positive bacteria.

## The FapR system as a biotechnological tool

In the last few years, a number of research groups have taken advantage of the unique properties of FapR to design and construct malonyl-CoA biosensors. Recently, a FapR-based malonyl-CoA sensor has been developed to detect changes of malonyl-CoA flux in living mammalian cells (Ellis and Wolfgang, 2012). After codon optimization, FapR from *B. subtilis* was fused to VP16, a viral transcriptional activator. The VP16 fusion converted FapR from a bacterial transcriptional repressor into a transcriptional activator in the absence of malony-CoA. The FapR operator sequence (*fapO*) was then multimerized and cloned upstream of a minimal promoter driving a reporter gene. This FapR-based malonyl-CoA biosensor was proven to be transcriptionally regulated by malonyl-CoA in mammalian cells and the reporter gene activity was demonstrated to be correlated with the intracellular levels of this effector molecule (Ellis and Wolfgang, 2012). This biosensor was then used to identify several novel kinases that when expressed in COS1 cells (a fibroblast-like cell line derived from monkey kidney tissue) promoted an increment of malonyl-CoA concentrations. In particular, it was shown that the expression of one of these kinases, LIMK1, altered both fatty acid synthesis and fatty acid oxidation rates. Thus, this simple malonyl-CoA responsive biosensor proved to be useful for the study of lipid metabolism in live mammalian cells and the identification of a novel metabolic regulator (Ellis and Wolfgang, 2012).

Two independent groups reported the development of a malonyl-CoA biosensor based on the FapR system of *B. subtilis* in the yeast *Saccharomyces cerevisiae* (Li et al., 2015; David et al., 2016). In both cases FapR was directed to the nucleus where it acted as a repressor on a synthetic promoter containing the FapR-operator site in optimized positions. The biosensors were validated and showed to reflect the change of intracellular malonyl-CoA concentrations. Both groups then used the malonyl-CoA biosensor to improve the production of the biotechnological valuable intermediate 3-hydroxypropionic acid (3-HP), which serves as the precursor to a series of chemicals, such as acrylates. Each group followed a different strategy to achieve this goal. Li et al. (2015) used the malonyl-CoA biosensor to screen a genome-wide overexpression library resulting in the identification of two novel gene targets that raised the intracellular malonyl-CoA concentration. Furthermore, they overexpressed the identified genes in a yeast strain carrying a bifunctional enzyme, *ca*MCR, from *Chloroflexus aurantiacus* that acts both, as an NADPH-dependent malonyl-CoA reductase and as a 3-hydroxypropionate dehydrogenase, converting malonyl-CoA to malonic-semialdehyde first and then to 3-HP. Interestingly, the authors found that the recombinant yeast strains producing higher amounts of malonyl-CoA showed over 100% improvement of 3-HP production (Li et al., 2015). Using a different approach, David et al. (2016) expressed the gene coding for *ca*MCR (*mcrCa*) under the control of the FapR-based biosensor. This self-regulated system gradually expressed the *mcrCa* gene depending on the available concentration of malonyl-CoA. Subsequently, in order to increase the malonyl-CoA supply for 3-HP production, the authors (David et al., 2016) implemented a hierarchical dynamic control system using the PHXT1 promoter to render FAS1 expression dependent on the concentration of glucose. FAS1 codes for the β-subunit of the fatty acid synthase complex in *S. cerevisiae*, while the α-subunit is encoded by FAS2. The expression of FAS1 and FAS2 is co-regulated, implying a coordinated up—or downregulation of the entire FAS system. Hence, when the external glucose concentration is low the PHXT1 promoter is repressed and FAS1 gene expression is downregulated, decreasing the consumption of malonyl-CoA in fatty acid biosynthesis. As a consequence, there is an increment in the intracellular malonyl-CoA concentration available for 3-HP production. Using this hierarchical two-level control and the fine-tuning of *mcrCa* gene expression, a 10-fold increase in 3-HP production was obtained (David et al., 2016).

Aliphatic hydrocarbons produced by microorganisms constitute a valuable source of renewable fuel so, in order to satisfy the global energy demand, high productivity and yields become essential parameters to achieve. Nowadays big efforts in microbial biofuel production are dedicated to build efficient metabolic pathways for the production of a variety of fatty acid-based fuels. In this regard, two studies were reported on the implementation of the FapR system in *E. coli*, which originally lacks the *fap* regulon, for the improvement of fatty acid production (Xu et al., 2014a; Liu et al., 2015). Malonyl-CoA, produced by ACC (Figure 1), is the rate limiting precursor for the synthesis of fatty acids. The *E. coli* ACC is composed of four subunits: a biotin carboxyl carrier protein, a biotin carboxylase, and two carboxyltransferase subunits. The overexpression of the genes coding for the ACC subunits improves fatty acids production but at the same time is toxic to the cells (Davis et al., 2000; Zha et al., 2009). To overcome this drawback, Liu et al. designed a strategy for increasing malonyl-CoA synthesis reducing the toxicity provoked by the concomitant *acc* overexpression (Liu et al., 2015). To this end, they built a negative regulatory system for the *acc* genes based on the ability of FapR to respond to the level of malonyl-CoA. Their goal was to promote a reduction in *acc* expression when malonyl-CoA levels were high and induce it when the malonyl-CoA levels were low. This required the design of a rewired system to create a negative feedback circuit. To this end, the *B. subtilis fapR* gene was cloned into *E. coli* using a low copy number plasmid under the control of a P_BAD_ promoter responding to arabinose. A FapR-regulated synthetic promoter (PFR1) was also constructed by inserting the 17-bp FapR operator sequence into two regions flanking the−10 region of a phage PA1 promoter. PFR1 was validated as a FapR-regulated promoter by analyzing the expression of a fluorescent protein under its control in response to different concentrations of malonyl-CoA (Liu et al., 2015). To complete the circuit, the *acc* genes were placed under the control of a LacI-repressive T7 promoter, PT7, and the *lacI* gene was placed under the control of PFR1. Hence, *acc* expression is initiated upon IPTG induction, producing malonyl-CoA. When malonyl-CoA is accumulated in this strain, the expression from PFR1 will turn on producing LacI, which in turn down-regulates *acc*, decreasing the malonyl-CoA synthesis rate. Using this approach, it was demonstrated that the negative feed-back circuit alleviated growth inhibition caused by either ACC overexpression or malonyl-CoA accumulation (Liu et al., 2015). In addition, this method was used for improving fatty acid titers and productivity and, in principle, could be extended to the production of other chemicals that use malonyl-CoA as precursor (Liu et al., 2015). Xu et al. (2014b) also constructed a malonyl-CoA sensing device by incorporating *fapO* into a hybrid T7 promoter that was shown to be able to respond to a broad range of intracellular malonyl-CoA concentrations, inducing the expression from the T7 promoter at increasing concentrations of the effector molecule. Interestingly, this group then discovered that the FapR protein could activate gene expression from the native *E. coli* promoter P_*GAP*_ in the absence of malonyl-CoA, that malonyl-CoA inhibits this activation, and that the dynamic range (in response to malonyl-CoA) can be tuned by incorporating *fapO* sites within the P_*GAP*_ promoter (Xu et al., 2014a). In order to improve fatty acid production, the genes coding for the ACC were then put under the control of the P_*GAP*_ promoter and the fatty acid synthase (*fabADGI* genes) and the soluble thioesterase *tesA*′ were placed under the control of the T7-based malonyl-CoA sensor promoter. Upon constitutive FapR expression, the resulting genetic circuit provided dynamic pathway control that improved fatty acid production relative to the “uncontrolled” strains (Xu et al., 2014a). Taken together, these studies highlight FapR as a powerful responsive regulator for optimization and efficient production of malonyl-CoA-derived compounds.

## Conclusions and perspectives

FapR is a global transcriptional repressor of lipid synthesis highly conserved in Gram-positive bacteria. Notably, the activity of this repressor is controlled by malonyl-CoA, the product of the first dedicated step of fatty acid biosynthesis, converting FapR into a paradigm of a feed-forward-modulated regulator of lipid metabolism. The activity of other well-characterized bacterial lipid regulators, like FadR of *E. coli* (van Aalten et al., 2001) or the TetR-like *P. aeruginosa* DesT (Miller et al., 2010), is feedback controlled by the long-acyl chain-end products of the FASII pathway (Zhang and Rock, 2009; Parsons and Rock, 2013). The EBDs of these proteins, frequently exhibit an α-helical structure with a relaxed specificity for long-chain acyl-CoA molecules, possibly because helix-helix interactions are permissive enough to constitute a platform for the evolution of a binding site for fatty acids of diverse chain lengths (Albanesi et al., 2013). In contrast, the feed-forward regulation mechanism of the FapR repressor family, which implies the recognition of the upstream biosynthetic intermediate malonyl-CoA, requires a high effector-binding specificity. In FapR, this high specificity is achieved by confining the charged malonyl group into a quite rigid internal binding pocket, and may be the reason why the “hot-dog” fold was recruited for this function (Albanesi et al., 2013). It is important to note that organisms using the FapR pathway could also count on a complementary feed-back regulatory loop operating at a biochemical level, for instance by controlling the synthesis of malonyl-CoA (Paoletti et al., 2007). If this is proven, it would imply that FapR-containing bacteria finely tune lipid homeostasis by feed-back and feed-forward mechanisms, as it indeed happens in higher organisms ranging from the nematode *Caenorhabditis elegans* to humans (Raghow et al., 2008).

Human health and life quality have significantly improved with the discovery of antibiotics for the treatment of infectious bacterial diseases. However, the emergence of bacterial resistance to all antimicrobials in clinical use (Levy and Marshall, 2004; Davies and Davies, 2010) has caused infectious bacterial diseases to re-emerge as a serious threat to human health. This scenario highlights the need to develop new strategies to combat bacterial pathogens. FapR controls the expression of many essential genes for bacteria not only involved in fatty acids but also in phospholipid synthesis. It has been experimentally shown that the presence of mutant variants of FapR unable to bind malonyl-CoA result lethal for bacteria (even in the presence of exogenous fatty acids), as the regulator remains permanently bound to DNA impeding the expression of its target genes. These results and the existence of FapR in important human pathogens validate FapR and lipid homeostasis as interesting targets for the search of new antibacterial drugs. With another perspective, the high specificity of FapR for malonyl-CoA has allowed for the development of *in vivo* malonyl-CoA sensors in diverse organisms that originally lack FapR and the *fap* regulon. These sensors have been shown to function in mammalian cells, in yeast and in bacteria responding accurately to the intracellular variations in the concentration of malonyl-CoA. The different FapR-based-malonyl-CoA biosensors were constructed following alternative strategies and used with a broad range of purposes focused on biological processes involving malonyl-CoA, including signaling mechanisms and metabolic engineering. Malonyl-CoA is the precursor of many industrial-valuable compounds like fatty acids, 3-hydroxypropionic acid, polyketides, and flavonoids, since they can be used as or converted to biofuels, commodity chemicals, fine chemicals, and drugs. Due to the success in the implementation of the FapR-based biosensors to improve the productivity and yields of the production of several malonyl-CoA-derived compounds, it is expected that new biotechnological applications of the FapR system emerge in the short term.

## Author contributions

DA and DdM conceived and wrote this review.

### Conflict of interest statement

The authors declare that the research was conducted in the absence of any commercial or financial relationships that could be construed as a potential conflict of interest.
